# Which Are the Main Factors Influencing Corporate Social Responsibility Information Disclosures on Universities’ Websites

**DOI:** 10.3390/ijerph18020524

**Published:** 2021-01-10

**Authors:** Raquel Garde Sanchez, Manuel Pedro Rodríguez Bolívar, Antonio Manuel López Hernandez

**Affiliations:** Department of Accounting and Finance, University of Granada, 18071 Granada, Spain; manuelp@ugr.es (M.P.R.B.); alopezh@ugr.es (A.M.L.H.)

**Keywords:** online disclosure, corporate social responsibility, USA, higher education

## Abstract

Universities are now becoming more active in the field of Corporate Social Responsibility (CSR). Nevertheless, they do not appear to be granting the same degree of importance to the dissemination of these activities. This article analyses the voluntary corporate social responsibility information disclosed by leading USA universities. We created several indexes of corporate social responsibility information disclosure and examined main universities’ characteristics that affect corporate social responsibility disclosure by these entities. The findings obtained show that the universities are strongly committed to the dissemination of corporate social responsibility information, and that a university’s size, affiliation, public/private status and ranking position are the factors most significantly affecting its online disclosure of general corporate social responsibility information. These findings could be useful for university administrators, especially those in public universities, highlighting the importance of developing and supporting policies and incentives to promote CSR disclosure and thus attract new students and meet social expectations about the ethical behaviour of universities.

## 1. Introduction

Corporate social responsibility (CSR) has been defined as “the responsibility of enterprises for their impacts on society […] to integrate social, environmental, ethical, human rights and consumer concerns into their business operations and core strategy in close collaboration with their stakeholders [1]. This issue is of growing importance in mainstream businesses and in the academic world, as institutions seek to promote a better and more sustainable world [2].

In response, universities are now becoming more active in the field of corporate social responsibility [3,4]. Universities, as leaders of socio-economic change, play a prominent role in overcoming the social, economic and environmental challenges faced by society [5,6]. They are becoming increasingly aware of the negative and/or positive impacts of their actions in their own environment and of the need to take a leading part in positive actions, as a model of ethical behaviour to society.

Among the corporate social responsibility strategies commonly adopted by firms, corporate social responsibility disclosure is considered the most important means of communication to stakeholders regarding corporate social responsibility activities [7]. Corporate social responsibility reporting is defined as the process of communicating the social and environmental actions of organisations to particular interest groups within society and to society at large [8].

By publishing corporate social responsibility information, universities can provide the information demanded by stakeholders, increase transparency, enhance their reputation and legitimacy, facilitate benchmarking with other universities and support corporate information and control processes [9]. This information would help universities to justify their activities to a wide range of stakeholders by providing a higher level of CSR disclosure regarding a number of social and environmental issues, going beyond a simple description of the economic environment facing the institution [10].

However, despite extensive academic and corporate interest in the subject, a review of the main journals addressing aspects of corporate social responsibility shows that little research has been undertaken to analyse the crucial role of universities’ disclosure of CSR information [11]. Moreover, few studies have sought to identify the underlying determinants of organisations’ online disclosure of social and environmental information [8]. Universities are no exception to this knowledge gap, and therefore our research is intended to make a useful contribution to the literature in this respect.

According to stakeholder theory, variables such as size, affiliation, private-public status, age and ranking position can influence the online disclosure of CSR information. The degree of CSR disclosure by universities is positively associated with their age and size. Larger and older universities usually address a wider audience, are subject to the assessment of a greater number of stakeholders and, therefore, have a greater impact on society.

In the other hand, universities with some faculty regarding CSR implies that universities that have faculties or departments which address aspects of CSR are usually more committed to the disclosure of this type of information.

In the competitive environment, universities increased competition to attract students. We find that private universities are more aware of applying criteria of social responsibility they can obtain a competitive advantage and maintain good relations with stakeholders and satisfying expectations [5,6]. These universities must invest resources and effort to distinguish themselves from public universities, not only concerning the quality of the education provided and of the research carried out, but also in other essential questions such as the promotion of ethical behaviour and the application of the principles of social responsibility.

By last, the online disclosure of CSR information could represent an element by which universities can be differentiated in terms of the ranking awarded for their quality of education and research.

Therefore, the aim of this paper is to examine the voluntary corporate social responsibility information disclosed by leading USA universities and to analyse how the main characteristics of universities—their size, affiliation, private-public status, age and ranking position, among other aspects –can influence the online disclosure of CSR information.

The contribution of this study is twofold. On the one hand, we present a new approach to evaluating the voluntary corporate social responsibility information disclosed by universities. A model is proposed with which to measure the degree of disclosure of this type of information and, therefore, each university’s commitment to CSR and its subsequent disclosure to stakeholders. On the other hand, the paper seeks to determine the variables or factors that may influence this information disclosure. Thus, the proposed evaluation model can be used to identify best practices and to reveal which models or types of universities are most strongly committed in this respect. This knowledge will facilitate the adoption of policies to meet social expectations about the ethical behaviour of universities, and at the same time help make them more competitive.

Taking into account the above considerations, Section 2 describes the theoretical framework employed, Section 3, the formulation of hypotheses, Section 4, the research design and method applied, Section 5, the results obtained, Section 6, the discussion of these findings and Section 7, the main conclusions drawn.

## 2. Theoretical Framework

The present paper is based on the stakeholder theory, which has been applied to the disclosure of CSR information. Stakeholder theory is an important reference in corporate social responsibility disclosure research, and many studies have used it to explain the organisation–society relationship [12,13]. In fact, stakeholder theory has become a fundamental tenet in the dissemination of CSR information, explaining the relationship between stakeholders and the information they receive [14], addressing the expectations of specific groups within society [15,16] and predicting corporate actions in response to stakeholder pressure, related to power dependence or claims of legitimacy [17].

According to stakeholder theory, the long-term success and survival of an organisation depends on the support it receives from stakeholders. Accordingly, the disclosure of corporate social responsibility information is considered a vital aspect of managing their needs, expectations and demands, and of balancing potential conflicts among them [18]. Thus, CSR information disclosure reflects a strategic position adopted by the organisation towards the social demands faced.

The demand for corporate social responsibility disclosure has been driven by the increasing prominence and interventionism of stakeholders [19]. In response, firms provide CSR disclosure seeking to legitimise their behaviour, producing a positive impact on stakeholders and on society’s perceptions [20]. The perceptions and expectations of stakeholders is an important factors driving the advancement of CSR-related practices by organizations. The increase in social sensitivity of society towards the behavior of companies, due to the fact that the different business scandals have occurred, show a change in the perception that citizens have about companies, causing a lack of confidence in these companies. Thus, this lack of trust highlights the necessary transparency in business for the construction of trust [5,6,12].

The presentation of corporate social responsibility information is a means of promoting dialogue with stakeholders [14]. This theory is of special relevance in the public sector, since public utilities must often address a wide range of stakeholders, many of whom have a legitimate interest in receiving information on an organisation’s financial, environmental and social issues. Thus, universities should take into account the needs of their stakeholders and the impact on them of the information disclosed. Indeed, when a public organisation has a large number of stakeholders (mainly composed of citizens and society in general), the pressure on it to disclose additional information with regard to issues of visibility and accountability is much higher than for privately owned firms [21].

## 3. Factors Influencing CSR Information Disclosure by Universities: Study Hypotheses

This paper focuses on the following university-related variables: size, affiliation, public versus private status, age and ranking position. These specific variables were studied because they have been justified in the theoretical framework analyzed and they are the main ones considered in previous studies of information disclosure, both in general [22,23,24] and by universities in particular [25,26].

Although some of the variables in our model have been proposed in other research studies concerning universities [26], we analyse different variables and apply the findings in a different context. The differences in legal and political contexts that are embodied in the organisational structures of government are significantly influenced by cultural differences between countries, giving rise to different forms of administration and management in the public sector.

### 3.1. Size

Organisations are subjected to numerous pressures to disclose CSR information [27,28,29]. According to stakeholder theory, when a public organisation has a large number of stakeholders, the pressure on it to disclose additional information with regard to issues of visibility and accountability is much higher [21]. Larger companies, thus, will disclose more CSR information in order to meet society’s expectations of them. Moreover, the variable *size* has frequently been used to explain the extent to which corporations are pressured to disclose information [23]. From an empirical standpoint, several studies have found a positive relationship between organisation size and the disclosure of CSR information [24,27].

Such disclosure is sometimes costly and so it is larger organisations that usually disclose most information [27]. Furthermore, it is the larger organisations which exercise most power in society, are most visible and, therefore, are most exposed to public scrutiny, with their performance being analysed by broad groups of stakeholders [24]. This factor, too, motivates these organisations to publish larger volumes of information, in order to improve their image and reputation.

This positive relationship has also been observed in the university sector, by studies such as those by Gordon et al. [25] and Gallego et al. [26], who found that university size has a positive influence on the amount of information disclosed. In view of these considerations, we propose the following hypothesis:

**Hypothesis 1** **(H1):** *There is a positive relationship between a university’s size and the CSR online information disclosed on their website*.

### 3.2. University Affiliation

In the private sector, the delegation of functions and tasks to various departments has produced specialisation, with each one being headed by professionally qualified management experts. Therefore, when reports from different areas are prepared and published, the process is supported by the heads of each section of the organisation and their joint efforts enable a comprehensive overview of the business. Previous research suggests that organisations with CSR-related departments are more likely to disclose information in this respect [29,30].

According to stakeholder theory, organisations with various CSR-related departments are usually more closely involved with social and environmental issues and publish a greater amount of information, seeking thus to meet the needs of a wide range of stakeholders [31].

A university is composed of various schools and faculties offering many different degrees and curricula. Extrapolating the idea of a business focus in this area, we believe that universities that have faculties associated with CSR will possess qualified personnel who may intervene in decisions and in planning actions that refer to CSR; equally, such personnel may provide a more complete understanding and facilitate the dissemination of CSR information. Accordingly, the following hypothesis is proposed (see Table 1):

**Hypothesis 2** **(H2):** *Universities with faculties related to the field of CSR will disclose more CSR online information on their website than those in which no such faculties are present*.

### 3.3. Private vs. Public Universities

The university system in many countries, and especially in the USA, is characterised by the coexistence of public and private universities [25]. Although these two types of institution have many features in common, they are fundamentally different in terms of their funding. Thus, whilst private universities largely depend on the tuition fees paid by students and on private donations [25], public universities are mainly funded by the State.

Falling student numbers in recent years, and the consequent reduction in State funding [25], has led to increased competition to attract students. In this respect, private universities are at a disadvantage since the tuition fees paid by students are their main source of funding [25], which explains their strong sense of marketing and their need to act according to business criteria. Consequently, these universities must invest resources and effort to distinguish themselves from public universities, not only concerning the quality of the education provided and of the research carried out, but also in other essential questions such as the promotion of ethical behaviour and the application of the principles of social responsibility. According to previous research in the private sphere [32], this could enhance their reputation and public image, making them an attractive option for more students. Besides, according to stakeholder theory, a greater disclosure of CSR information could help private universities better respond to their stakeholders’ needs, thus creating a differentiating factor and providing a competitive advantage.

Therefore, apart from universities’ teaching and research quality, which is an essential element in their reputation, socially responsible behaviour by universities and how this behaviour is reflected through informational transparency could produce a competitive advantage in the higher education market, which in turn could provide universities with greater legitimacy in their relationship with stakeholders. The use of new information technology, and specifically, the internet, has proved to be a very important tool in this process [25,30].

For this reason, it is expected that private universities, in view of the strong competition they face to obtain financial resources and of the competitive advantage that might be gained from the disclosure of CSR information, will present higher levels of disclosure of this information on their official websites. Therefore, the following hypothesis is proposed (see Table 1):

**Hypothesis 3** **(H3):** *Private universities disclose a larger volume of CSR online information on their website than public universities*.

### 3.4. Age

In previous research, the variable *age* has been considered an influential factor in information disclosure. In the present study, it is used to refer to the time elapsed since the university was established [26], in the view that universities which have been in existence for longer will have been subject to greater scrutiny by stakeholders. According to stakeholder theory, an organisation’s existence depends on its ability to integrate stakeholders’ expectations into its business strategy, because stakeholders provide resources that are essential to the organisation’s successful functioning and survival [33]. Therefore, universities must respond to stakeholders’ needs and demands regarding CSR, and this responsibility increases in line with the time that the organisation has been in existence, because older organisations will have had more time and gained more experience in the development of all types of policies and their subsequent disclosure. Moreover, these policies will have been made public for longer.

It has been observed that the degree of CSR disclosure by universities is positively associated with their age [26], and thus older universities disclose more information than newer ones [34]. This is not surprising, since older universities have had longer to develop information of all kinds and to disclose it to their different stakeholders. Therefore, the following hypothesis is proposed:

**Hypothesis 4** **(H4):** *Older universities disclose more CSR online information on their website than younger universities*.

### 3.5. Ranking Position

According to Cooke [35], when a firm is listed on a foreign stock exchange as well as that of its country of domicile, it will disclose more detailed information since it will need to observe the disclosure rules of at least two exchanges, and so it will attract more analyst coverage [24]. Numerous studies have reported finding a significant positive relationship between international listing status and the level of voluntary CSR information disclosure [24,36]. 

Extrapolating this variable to consider the field of universities, good corporate results are reflected, in this case, as the achievement of a favourable position in rankings of teaching and research quality. Therefore, according to stakeholder theory, universities, too, will provide more information in order to underpin their position within the market [24].

The introduction of competitive financing mechanisms in higher education has led to the development of new strategies for attracting students. Highly rated universities tend to be characterised by inter-organisational linkages, i.e., voluntary ties with third parties, such as academic spin-offs and other informal mechanisms of technology transfer [37].

Acceptance of the relationship between legitimacy and information disclosure has led to the creation of university classification systems [38]. These classifications receive considerable attention among the university community and society in general. They provide an index of the quality of the higher education system, are indicative of an institution’s reputation and represent its degree of competitive advantage in areas such as attracting students and retaining government financial support [39].

Universities with greater legitimacy are more closely scrutinised by stakeholders and assign a high priority to the outcome of the evaluation process underlying the rankings system. The lack of such recognition would be detrimental to the legitimacy of the university [39].

Prior research indicates that the universities reputed to provide the highest quality education and research are the most likely to disclose CSR information online [3]. Thus, the online disclosure of CSR information could represent an element by which universities can be differentiated in terms of the ranking awarded for their quality of education and research. In this respect, according to McNamara [40], the world’s most prestigious universities are expected to beat the forefront of great movements for social change (see Table 1). In consequence, the following hypothesis is proposed:

**Hypothesis 5** **(H5):** *The universities offering the highest quality education and research are the strongest proponents of the disclosure CSR online information on their website*.

## 4. Methodology

### 4.1. Sample

Numerous universities have shown an outstanding commitment and pioneering spirit in developing corporate social responsibility policies, especially in European countries [41], including these concerns in their management, academic and research functions and in the university’s mission/vision [42]. They are strongly committed to corporate social responsibility, in the scale and impact of their actions, in their tradition and in their social influence. Universities undertake corporate social responsibility actions both to establish the legitimacy of their operations and to attract students, fees and subsidies [43]. Nonetheless, in order to obtain or maintain this legitimacy, they must not only take actions but also inform society at large about these actions [44]. It has been shown that one of the main strategies applied by organisations to gain legitimacy for their actions is to align stakeholders’ perceptions and expectations, by means of information disclosure [10].

In this analysis of best practices in CSR voluntary communication, our study sample was drawn from USA universities. Although of course many universities worldwide publish CSR reports, those in the USA are the most active in the online disclosure of CSR-related information [5]. The USA has the second largest number of higher education institutions in the world and the largest number of students in higher education [45], which makes this context very suitable for obtaining solid research findings. Moreover, USA universities need to satisfy a large group of stakeholders in order to legitimise and continue their activities and have long been distinguished by their commitment to “service to the community” [46].

Our selection of universities for analysis has some limitations. First, it only includes those which implement corporate social responsibility actions and report their policies online. Therefore, the present study does not address universities that, while committed to corporate social responsibility, do not disclose their commitment or, even if they do, use other means, such as printed materials (posters, brochures, magazines, etc.). Nevertheless, we believe the sample considered is appropriate for the goals of this study, i.e., to analyse the factors influencing online transparency on CSR-related issues.

Secondly, the 154 leading USA universities included in our sample were selected according to the Academic Ranking of World Universities (ARWU), more commonly known as the Shanghai ranking. Although this ranking has been questioned and its methodological limitations highlighted [47], the ARWU is generally agreed to be acceptable in terms of objectivity and comprehensiveness [48]. The ARWU index of world universities, based on academic quality and overall excellence, has been used in numerous previous studies [49]. Furthermore, it is one of the instruments most widely employed in research studies for measuring institutional quality [50]. Therefore, in order to obtain an appropriate sample for this study, we selected all the USA universities in the top 500 of the Shanghai ranking. The final sample thus obtained consisted of 154 universities, of which 105 (68.18%) were public and 49 (31.82%) were private (see Appendix A).

### 4.2. Empirical Model

In this section, we derive a model to represent the influence of the above factors on CSR information disclosure (see Table 1). The model was tested empirically by multiple linear regression, estimated by ordinary least squares (OLS) [26,27,44,51].

MODEL: the approach adopted in the empirical analysis is summarised by the following general form of the model:

GSRD_i_ = α_0_ + β_1.1_ Size_i_ + β_2.1_Affiliation_i_ + β_3.1_UniPriv_i_ + β_4.1_ Age_i_ + β_5.1_ Rank_i_+ ε_i_

ESRD_i_ = α_0_ + β_1.2_ Size_i_ + β_2.2_ Affiliation_i_ + β_3.2_ UniPriv_i_ + β_4.2_ Age_i_ + β_5.2_Rank_i_+ ε_i_

NSRD_i_ = α_0_ + β_1.3_ Size_i_ + β_2.3_Affiliation_i_ + β_3.3_UniPriv_i_ + β_4.3_ Age_i_ + β_5.3_Rank_i_+ ε_i_

SSRD_i_ = α_0_ + β_1.4_ Size_i_ + β_2.4_Affiliation_i_ + β_3.4_UniPriv_i_ + β_4.4_ Age_i_ + β_5.4_Rank_i_+ ε_i_

DSRD_i_ = α_0_ + β_1.5_ Size_i_ + β_2.5_ Affiliation_i_ + β_3.5_UniPriv_i_ + β_4.5_ Age_i_ + β_5.5_Rank_i_+ ε_i_

where GSRD_i_; ESRD_i_; NSRD_i_; SSRD_i_; DSRD_i_ are the dependent variables in every model referring to each USA university;

β _i.j_ are the coefficients of the explanatory variables for each evaluation model proposed (GSRD_i_; ESRD_i_; NSRD_i_; SSRD_i_; DSRD_i_) and the variables are defined as in Table 1.

Table 1 shows the explanatory variables examined to test the research hypotheses. The data on these variables were obtained from each university’s website. The results obtained show that the variables derived from the items described present a high degree of internal consistency, as shown by the Cronbach’s alpha value obtained, which in all cases exceeds 0.7 [52].

Many studies have used total assets, sales or market capitalisation to measure firm size. However, as Gordon et al. [25] observed, market capitalisation is not a measurable value for universities. An appropriate alternative measure of size could be logarithm of the number of students [26] (see Table 1).

The number of students, as a variable reflecting the size of the university, is a significant factor. Students are, in fact, the institution’s main stakeholders and the major consumers of its resources [26]. Thus, previous studies have noted the growing concerns about CSR among university stakeholders, including students, faculty and parents (see Fonseca [53], for universities in Canada, and Yuan [54] on the situation in China) (see Table 1).

*Affiliation* is a dummy variable which takes the value 1 if the university has a faculty related to CSR, and 0 otherwise. *Public versus private status* is a dummy variable which takes the value 1 if the university is a private institution and 0 otherwise. The *Age* variable is measured by logarithm of the number of years elapsed since the university’s foundation, and the ranking is the university’s position in the Shanghai ranking (see Table 1).

The dependent variables were obtained from the analysis of items in the disclosure index for the websites (see Table 2 and Table 3). However, no standard model has yet been established for the evaluation of CSR reports or CSR disclosure, although work is being done in this area, for example, by the University Leaders for a Sustainable Future (ULSF).

The Global Reporting Initiative (GRI) Sustainability Guidelines [55,56] were taken into account for the selection of the model items. Based on these guidelines, a search was made of a sample of universities in order to check the information disclosed about these aspects and thus ensure that the information analysed corresponded to the specific area of institutions of higher education.

Specifically, the index of general CSR information disclosure (GSRD), taking into account the structure of the GRI guidelines, is divided into five sections: 1. Vision and Strategy; 2. Profile; 3. Governance Structure and Management Systems; 4. GRI Content Index; 5. Performance Indicators [55,56,57].

The GRI guidelines are organised in terms of economic, environmental and social performance. These items configure the sub-indices related to specific CSR information (ESRD, NSRD, SSRD). For the purposes of the present study, these items were adapted according to the specific nature of the information provided by the universities.

The GRI guidelines are among the most complete tools available with which to assess CSR information disclosure. Although the guidelines were not intended specifically for this purpose [58], they provide an excellent instrument for evaluating university CSR reports [57,59]. A limitation of the guidelines is that they do not address indicators related to the incorporation of CSR-related issues in research activities and study plans, or areas such as green buildings and food services, among other areas relevant to colleges and universities. However, as argued by Lozano [57] and others, the guidelines could and should be amended and supplemented to include these characteristics. In the present study, to fill this gap, the content analysis framework used includes indicators drawn from campus CSR assessment tools and from previous research [57,60].

In their core competences, i.e., education and research, universities differ substantially from corporations. Therefore, for the GRI to be suitable for our field of study, it must include the education dimension. In this respect, various universities and many researchers and educators have been collaborating with the University Leaders for a Sustainable Future (ULSF) team to develop a standardised version of the GRI for universities [57]. Taking into account these considerations, the ULSF draft proposals were adapted to obtain the item that addresses the education dimension within the present area of study (DSRD).

In summary, a model was created to evaluate online CSR information disclosure by universities, considering the economic, environmental and social dimensions established in the GRI guidelines report and adding, for the case of universities, the educational dimension proposed by the ULSF. Based on these items, the model was developed to ensure its appropriateness for the specific field of universities.

We believe this model extends and enhances the literature on CSR information disclosed by universities. The model we present was created in a multi-stage process. First, various search engines were used to determine the CSR information currently being disclosed online by universities, taking the total population as the study sample. Different types of data were analysed to assess the sustainable practices used, by examining the following sources: (i) university web pages (ii) annual reports; (iii) CSR reports (or sustainability reports as they are termed by some universities in accordance with the GRI guidelines and which include CSR information). Consequently, meetings were held to discuss the results obtained, following which a draft model was proposed, summarising the main aspects of the information disclosed. No restrictions of country or region were imposed regarding the universities considered.

This process gave an initial approximation of the model to be analysed. It was then completed in accordance with the guidelines issued by international bodies, such as the GRI, and taking into account previous research in this area (by Lozano, for example), with particular respect to the university context.

This evaluation model was then applied to the USA universities selected for analysis. The data required for this were obtained from the universities’ main channels of communication, i.e., their web pages (generally in HTLM format) or CSR reports (in PDF format).

Thus, the information on the dependent variables was obtained after a detailed analysis of CSR reports of the universities in the sample, in the view that these reports constitute an effective mechanism of control and communication [59]. We also report the information that the universities provide on their websites and in financial and annual reports.

Regarding the score assigned to each of the questions included in our proposal for the assessment of CSR information disclosure, and taking into account previous approaches [22], we opted for a binary dichotomous scoring system (0/1), reflecting the absence or presence of each item on the website or in the CSR report (see Table 2 and Table 3). When the items contained several sub-indices, their scores were distributed equally. This method was adopted in order to reduce the degree of subjectivity, in a scoring system for which there are no explicit, predefined rules [61]. Thus, the same value is awarded to each item when the aspect being analysed is described by various items [62] (see Table 2 and Table 3).

During April and May 2020, we examined the websites of the universities in our sample to obtain the information required. To ensure objectivity, the process was carried out separately by each of the three authors, who subsequently discussed the results and reached a consensus. If there were any significant discrepancies, the websites were examined again by all three authors. In the following months, we proceeded to the statistical analysis and final writing of the paper.

## 5. Results

### 5.1. Descriptive Results

Table 4 shows the descriptive statistics obtained for the dependent variables, expressed as the mean, median, standard deviation, frequency and percentage, thus presenting the general and specific content of the CSR information provided on the university websites.

Of the items included in the GRI recommendations, those which are least often disclosed by the universities in our sample are the stakeholders’ profiles and the performance indicators for the different dimensions of CSR (mean values of 0.01 and 0.07and frequencies of 0.65% and 6.64%, respectively). The items most commonly reported (outlook on CSR, centralised/decentralised information and table of contents) are provided by nearly 50% of the universities.

The universities examined are more committed to the provision of specific information, on their websites or in annual reports. In this respect, the highest values recorded were for the disclosure of economic or social information (70.65% and 70.37% respectively). High values were observed for economic information because it is usually published in the institution’s financial reports. Social information, on the other hand, tends to be more dispersed, on the website or within the annual report. Almost all items produced values close to 80% except those regarding equal opportunity (47.40%), the statement of integrity (14.29%) and the university’s code of conduct (57.14%) (see Table 4).

All these universities provide environmental information, but they only achieve just over 50% of the total score possible. The items most commonly disclosed refer to energy, waste management and recycling, while those least often published refer to sustainable food and fair trade. With respect to the educational information contained in these universities’ disclosure of CSR, the disclosure of voluntary service is the most highly valued item (81.82%), and the least, the description of research topics relevant to CSR (63.63%) (see Table 4). The present study focuses on the items that these universities currently provide online with respect to economic, environmental, social and educational information. Hence, our content analysis of these areas produced high values even though the standard deviations obtained do not indicate non-homogeneity.

### 5.2. Testing the Model

We next consider the influence of certain factors on the universities’ online disclosure of general and specific CSR information. Table 5 shows the details of the variables analysed.

The comparison of typified predicted values versus typified residuals revealed the presence of random features, and therefore there were no problems of heteroscedasticity or non-linearity; consequently, we accept the hypotheses of linearity and of equality of the variances. None of the variance inflation factors not reported exceeded the critical value of 10 and thus multicollinearity is not a serious problem in this study (3.176 Size, 1.300 Affiliation, 1.334 UniPriv, 1.205 Age, 1.435 Ranking). In view of these considerations, after having confirmed that the above statistical assumptions were met, and the validity of the model thus endorsed, we decided to use the OLS method, given its simplicity and ability to test the hypotheses.

With respect to the *size* variable, the universities analysed in this study have an average of 4.19 students (measured through logarithm of total number of students). 38% have CSR-related faculties or schools, and their average age is 2.09 years (measured through logarithm of number of years since the foundation year).

According to the results of the empirical analysis of our model (see Table 6), the models that analysed the explanatory factors of the disclosure of general and specific CSR information reflected a low explanatory power (16.6% General, 10.1% Economic, 22.5% Environmental, 46.2% Social and 13.3% Educational) with a confidence level of 99% (*p* < 0.001) for the general, environmental and social information and with confidence levels of 95% (*p* < 0.05) and 99% (*p* < 0.1) for educational and economic information, respectively. These results suggest that future studies should examine other variables that may have a greater influence on the online disclosure of CSR and specific information by universities.

Analysis of the factors that influenced our model, particularly regarding GSRD, the statistically significant variables were the universities’ *size*, *affiliation, public-private status* and *ranking position*.

For environmental information (NSRD), the variables *size* and *ranking position* all have a positive influence on the online disclosure of environmental information by the universities in our sample (β = 0.301 and 0.326, respectively) at 95% significance.

For economic information disclosed online (ESRD), the only significant variable was size and *age* (β = 0.181 and 0.176, respectively), while for educational information (DSRD), the only significant variable was *affiliation* (95%, β = 0.283). Finally, we recorded an inverse association between the social information disclosed online (SSRD) and the public-private status of the university (β = −0.129) and positive with respect *ranking position* (β = 0.657).

Overall, therefore, the results obtained by the model, for our sample data, support hypotheses H1, H2, H3 and H5, indicative of positive relations between the size, affiliation, private status of universities and ranking position in terms of the online disclosure of general CSR information. Furthermore, our results support H1 and H5 in relation to the disclosure of environmental information; H1 and H5 on economic information; H3 and H5 on social information and H2 on educational information. In every case, the signs obtained were consistent with those expected.

## 6. Discussion

The aim of the present study is to advance stakeholder theory [15] by showing that universities in the USA make use of the online disclosure of CSR information in order to meet their stakeholders’ expectations and interests. Moreover, by disclosing this type of information, universities enhance their transparency and present greater accountability to society.

Therefore, the main theoretical implications of this study are related to stakeholder theory. The provision of CSR information by universities contributes to the public good by adding value to the information supplied [63]. Universities that use ICTs to disclose information respond thereby to the demands of society and encourage greater interaction with it [23].

However, although the research findings presented in this paper show that universities are making use of these channels and are improving their provision of CSR information, the outcome falls short of our expectations. Thus, greater awareness among universities of the importance of communicating CSR information is still necessary. Improving their performance in this regard would contribute to universities’ fulfilling their duty of accountability to stakeholders, and favour the development of benchmarking procedures which, in turn, would help universities gain competitiveness in their market.

Our results show that, in general, the universities examined are more inclined to disclose information of an economic and social nature, perhaps because these aspects have a greater trajectory of disclosure through the annual reports.

Another aim of our study was to identify the factors that influence CSR information disclosure. With respect to disclosure on CSR in general, numerous factors have a significant positive impact, including university size, affiliation, private-public nature and ranking. According to stakeholder theory, larger universities usually address a wider audience, are subject to the assessment of a greater number of stakeholders and, therefore, have a greater impact on society [24,25,26,27]. These universities must seek to legitimise themselves in the eyes of their stakeholders in order to maintain their social approval and hence survive. This goal, logically, is shared by private universities, whose main objective is to attract sufficient numbers of students to ensure their own funding, and which do so by seeking to project a favourable image, via a high level of information disclosure. The internet is of fundamental importance in this respect, enabling universities to promote themselves both nationally and internationally [26].

Universities that have faculties or departments which address aspects of CSR are usually more committed to the disclosure of this type of information. Highlighting this fact can raise awareness among universities of the importance of having qualified teachers in related subjects, which can subsequently be grouped into units, departments and faculties.

Furthermore, the universities in the highest positions in the rankings for quality of education and research disclosure differ significantly and positively, in terms of CSR information provided, from those in low-ranking positions.

In line with previous studies, we find that private universities are more aware of these considerations, because by applying criteria of social responsibility they can obtain a competitive advantage and thus improve their financial results (in terms of increased assets, resources and intangible capacities), whilst maintaining good relations with stakeholders and satisfying expectations [29,32].

Similar results were found regarding the factors that significantly influence the disclosure of environmental information, i.e., university size and ranking. However, this is not the case for other areas of information. Private universities are the most committed to the disclosure of social information, reflecting the importance granted by these universities to areas such as student funding, campus safety, diversity, equal opportunity and summer programmes. Private universities pay special attention to these aspects because they are considered of crucial importance in attracting future students. In the highly competitive situation currently facing universities worldwide, CSR disclosure is an intangible asset of considerable interest to universities, especially private ones, as students’ tuition fees constitute their main source of income [25].

The variable affiliation is most strongly associated with the disclosure of information on educational issues related to CSR. Thus, the more faculties, departments and personnel dedicated to the area of CSR, the greater the university’s commitment to social, environmental, economic and educational issues. Moreover, such a university will also present greater interest in education and research in this area and in the disclosure of related information.

The variable age has a positive influence on the disclosure of information of an economic nature. The publication of this type of information has a long history in universities, and therefore it is to be expected that older universities will be more committed to the disclosure of such information in the field of CSR.

These considerations should be communicated to public managers and university administrators responsible for designing strategies for the online disclosure of CSR information and for increasing awareness of its importance. This information is demanded by society as a whole and by future students in particular, who may rely on it in making their choice of university.

In fact, private universities and those ranked highly in terms of quality of education and research are aware of this, and often prioritise the online provision of CSR information in order to attract future students [30].

University managers could enhance their disclosure strategies, for example by creating more faculties and departments related to CSR. This would generate more training and awareness and ultimately promote the implementation of CSR policies and the disclosure of related information.

Finally, it should be noted that our research work has highlighted a set of variables that may affect the disclosure of CSR information according to previous literature. However, the values obtained by the R2 in each model have been low. Maybe, these variables may have been justified in the private sphere or in sectors other than higher education. The universities, perhaps because of their nature and connotations, need a more profound study to look for other variables that will have a greater influence on the dissemination of this information.

## 7. Conclusions

The findings presented in this research paper are of interest from various standpoints. On the one hand, we provide a new understanding of universities’ online disclosure of information on CSR-related topics and propose a model for assessing the disclosure of diverse aspects of CSR. On the other, we analyse the factors that may promote this information disclosure.

The CSR information disclosed by the universities in our sample corresponds to the items included in our evaluation model. The results obtained highlight the importance of online CSR information disclosure by universities, in each of the aspects considered: social, environmental and educational.

With respect to factors that may promote information disclosure, our findings show that, all of the variables considered are associated with the increased provision of CSR-related information. We find, therefore, that universities respond to the information needs of their stakeholders in their CSR disclosure policies. The findings of this paper could be useful for university administrators, especially those in public universities, highlighting the importance of developing and supporting policies and incentives to promote CSR disclosure and thus attract new students and meet social expectations about the ethical behaviour of universities. In addition, we call upon university policy makers to contribute to the global conversation with regard to CSR reporting and thus meet the information needs of all stakeholders.

In practical terms, university managers should take into account the importance of ensuring the presence of university teachers who are well informed of the role of CSR, who address this topic in the classroom, and who subsequently communicate their knowledge to society at large. Legislators and educators, in their respective fields, should take measures to encourage the formation of future human capital through teaching, research and the transfer of knowledge, thus increasing general awareness of social responsibility and its importance. Accordingly, governments should support the education system by allocating resources for CSR training, so that teachers can transmit this awareness to their students, who may subsequently put it into practical effect in the business world.

We show that larger universities are more aware of their inter-relationship with their environment and of the need to strengthen departments and faculties related to CSR, in view of the greater impact of these institutions on society. In future research, it would be interesting to examine whether the number of faculties in each university addressing aspects of CSR in their study programmes might be related to the level of CSR disclosure made, and to the strategic planning conducted by the institution or to the CSR-related policies adopted.

We believe it would be useful for these rankings to include, in addition, the impact made by CSR disclosure by universities, as an index of their quality.

In conclusion, our paper contributes to stakeholder theory by proposing a new model for CSR disclosure quality, which can be used to evaluate the disclosure of this information by universities, based on the need to meet stakeholders’ needs for CSR information. Furthermore, analysis of the factors involved in CSR information disclosure by universities could help and encourage university administrators to implement policies with which to meet stakeholders’ expectations regarding CSR disclosure by universities.

Universities are to consider CSR as a strategic and differentiating factor. This question has been shown to be of significant importance in attracting students to the institution. Like that, public managers and university administrators should encourage policies that favour the dissemination of CSR. One way to raise awareness of these issues is through training of their own staff. In this way, specific departments and sections involved in the management and dissemination of CSR can be created. This will lead to a greater awareness of these aspects which in turn will translate into the training of future business leaders.

As limitations to our article, we emphasise that it focuses on USA universities. The variables included in our analysis should be examined according to the context in which the study findings are expected to be applied. Obviously, in some ways the context of universities in Europe will be different from that considered in this paper. For example, it would be interesting, in future research, to include variables such as the prevailing culture or the funding system applicable to universities.

Another limitation is the way in which the proposed CSR online information disclosure model is evaluated, since this is done in terms of the absence/presence of the parameter in question, without taking into account its quality and quantity. Thus, future research efforts should address the items that obtained the highest scores, in order to obtain a more nuanced view, weighting the evaluation made in terms of the quality and quantity of the parameters considered.

As further areas of interest for future research, we recommend the identification and analysis of other variables that may affect universities’ policies on the disclosure of CSR-related information. For example, variables concerning internal management and organisational culture, such as the balanced scorecard system or that of corporate governance, could be added to the analysis. The adoption of such a complementary approach could help us achieve a better understanding of the functioning of universities and of their forms of organisation and management, thus facilitating comparison with other areas of management and with other countries. It would be useful in future research to analyse other measures in order to determine the influence of these variables on CSR reporting (for example, size could be considered by reference to the university´s budget. In addition, it would be interesting to study the influence of these variables in other contexts and countries, to observe their similarities and differences. Furthermore, another future research of interest would be to measure how the provision of CSR is also a consequence of more efficient universities [64] to carry out comparative studies between universities and countries in this regard. From the resulting conclusions drawn, further advances could be made in this area of knowledge.

Another potential area for study is that of the public policies employed in the state or region in which the universities are located and to determine whether greater financial support might enable more CSR-related research to be conducted. Finally, with respect to universities’ image and reputation, researchers should examine the profile of the students addressed and consider how new students might be attracted via the CSR information supplied.

One question that remains unanswered in the present study is that of whether the online disclosure of CSR information by universities could enhance commitment to CSR among corporations, as a result of the education and training in this respect of future managers and through the creation of social movements and CSR discourses within student organisations (taking into account that student organisations are known to be receptive to the discourse of CSR reporting). Accordingly, a line of interest for future study would be to analyse the models required to manage and properly channel the university information on CSR that is provided to stakeholders. This question may be of decisive importance in students’ choice of university and in shaping the prestige of the institution. In this respect, an aspect of evident importance is that the online information provided by universities is now a major factor in determining where students choose to study.

## Figures and Tables

**Table 1:** Main variables.

MODEL		
Variable	Definition	Hypothesis
GSRD	General CSR Online Disclosure	Dependent variable
ESRD	Economic Information Online Disclosure	Dependent variable
NSRD	Environmental Information Online Disclosure	Dependent variable
SSRD	Social Information Online Disclosure	Dependent variable
DSRD	Educational Information Online Disclosure	Dependent variable
Size	University size, measured through logarithm of total number of students	H1
Affiliation	Dummy variable which takes the value 1 if the university has some faculty regarding CSR, and 0 otherwise	H2
UniPriv	Dummy variable which takes the value 1 if the university is private, and 0 otherwise	H3
Age	Logarithm of number of years since the foundation year	H4
Ranking position	Position of the university in the Shanghai ranking	H5

Source: Own elaboration.

**Table 2:** Online disclosure of general CSR information.

GENERAL CSR Online Disclosure		*m*	
		GSRD = ∑g_i_ /5	
		*i* = 1	
Concept	Items	Score	Source
1. Statement of vision and strategy of the university on issues about social responsibility	(a) If main CSR commitments are disclosed.	0/0.5 based on the absence-presence of this item	
	(b) If the webpage or Sustainability Report includes a declaration on CSR from the governing body.	0/0.5 based on the absence-presence of this item	Adaptation by GRI Guidelines
2. Information about profile of stakeholders	(a) If the university webpage or the CSR/SustainabilityReport identify the stakeholders.	0/0.5 based on the absence-presence of this item	
	(b) If there is specific information about the informational needs of each group of stakeholders.	0/0.5 based on the absence-presence of this item	
3. Centralized or decentralized disclosure of CSR information by universities	(a) If the disclosure of CSR information is developed in a centralized way on the university webpage.	0/0.5 based on the absence-presence of this item	
	(b) If this disclosure is developed through dependent centres at said university.	0/0.5 based on the absence-presence of this item	
4. Data on performance indicators	(a) Economic indicators.	0/0.33 based on the absence- presence of this item	
	(b) Social indicators.	0/0.33 based on the absence- presence of this item	
	(c) Environmental indicators.	0/0.33 based on the absence- presence of this item	
5. Index of contents or a table to locate different elements of information about CSR	Provides the reader with an index or a table to locate different CSR elements.	0/1 based on the absence-presence of this item	

Source: Own elaboration.

**Table 3:** Online disclosure of specific CSR information.

Economic Information Online Disclosure		*m*	
		ESRD = ∑g_i_/5	
		*i* = 1	
Concept	Items	Score	Source
1. Customer (Students)	Information is disclosed about Student Income (Student aid and tution)	0/1 based on the absence/presence of this item	Adaptation by GRI Guidelines
2. Suppliers	Information is disclosed about Payments to suppliers	0/1 based on the absence/presence of this item	
3. Employees	Information is disclosed about Employee benefits expense (Salaries, wages, and employee benefits)	0/1 based on the absence/presence of this item	
4. Providers of capital	Information is disclosed about Sponsored, non for profit, auxiliary enterprises, Private gifts, grants, and contracts	0/1 based on the absence/presence of this item	
5. Public sector	Information is disclosed about State appropriations (federal government)	0/1 based on the absence/presence of this item	
**Environmental Information Online Disclosure**		***m***	
		**NSRD = ∑g_i_ /6**	
		***i* = 1**	
**Concept**	**Items**	**Score**	**Source**
1. Energy	Information is disclosed about the installation of systems that save electricity such as movement sensors, incandescent lightbulbs or other alternative sources of energy.	0/1 based on the absence/presence of this item	Adaptation by GRI Guidelines
2. Buildings and grounds	Information is disclosed about criteria for construction, renovation and rehabilitation of existing buidlings in line with “green criteria”.	0/1 based on the absence/presence of this item	
3. Purchasing management	Information is disclosed about the need to prioritize the purchase of reusable, ecological materials that require a minimum of packaging.	0/1 based on the absence/presence of this item	
4. Waste management and recycling	Information is disclosed about questions related to the promotion of the recycling of office material and solid waste providing recipients for articles such as paper, printer cartridges and batteries.	0/1 based on the absence/presence of this item	
5. Transportation	Information is disclosed about the creation of incentives for the university community to use public transport or alternative means of transport such as bicycles and bus.	0/1 based on the absence/presence of this item	
6. Food	Information is disclosed about fair trade and sustainable food through the provision of ecological products in campus cafés and shops.	0/1 based on the absence/presence of this item	
**Social Information Online Disclosure**		**m**	
		**SSRD = ∑g_i_/11**	
		***i* = 1**	
**Concept**	**Items**	**Score**	**Source**
1. Summer programs	Information is disclosed about a specific section about Continuing Education with summer programs	0/1 based on the absence/presence of this item	Adaptation by GRI Guidelines
2. Employment	Information is disclosed about the opportunity to search jobs in the University or outside	0/1 based on the absence/presence of this item	
3. Campus services/Student life	Information is disclosed about the specific section regarding club-organizations, sport and recreation, student affairs, housing and dining; student organizations and activities; shopping and others.	0/1 based on the absence/presence of this item	
4. Campus safety	Information is disclosed about the specific section about safety services	0/1 based on the absence/presence of this item	
5. Health services	Information is disclosed about the specific section about health services	0/1 based on the absence/presence of this item	
6. Scholarship	Information is disclosed about the Scholarship	0/1 based on the absence/presence of this item	
7. Equal opportunity	Information is disclosed about The Office of Equal Opportunity where the value of diversity is recognized and where equal opportunity is afforded for all.	0/1 based on the absence/presence of this item	
8. Diversity and equity	Information is disclosed about diversity and equity services for students	0/1 based on the absence/presence of this item	
9. Disability resources	Information is disclosed about disability resources	0/1 based on the absence/presence of this item	
10. Statement of integrity	Information is disclosed about statement of integrity		
11. Code of conduct	Information is disclosed about code of conduct	0/1 based on the absence/presence of this item	
**Educational Information Online Disclosure**		**m**	
		**DSRD = ∑g_i_/3**	
		***i* =1**	
**Concept**	**Items**	**Score**	**Source**
1. Academic	Information is disclosed about courses, seminars and conferences related to CSR.	0/1 based on the absence/presence of this item	Adaptation by ULSF proposed Educational Performance Indicators
2. Research	Information is disclosed about University research centers linked to CSR.	0/1 based on the absence/presence of this item	
3. Service	Information is disclosed about Volunteer services	0/1 based on the absence/presence of this item	

Source: Own elaboration.

**Table 4:** Descriptive Statistics dependent variables.

General CSR Online Disclosure	Mean	SD	Percentage
**General CSR Online Disclosure (GSRD)**	**0.31**	**0.18**	**31.43%**
1. Expression of the vision and strategy of the university in CSR subjects	0.45	0.34	45.13%
2. Information on the profile of stakeholders	0.01	0.06	0.65%
3. Centralized or decentralized disclosure of SR information by Universities	0.49	0.29	48.90%
4. Data on performance indicators	0.07	0.17	6.64%
5. Index of contents or a table to locate different elements of CSR information	0.56	0.5	55.84%
**Specific CSR Online Disclosure**	**Mean**	**SD**	**Percentage**
**Economic Information Online Disclosure (ESRD)**	**0.71**	**0.42**	**70.65%**
1. Customer (Students)	0.74	0.44	74.03%
2. Suppliers	0.56	0.5	55.84%
3. Employees	0.73	0.44	73.38%
4. Providers of capital	0.75	0.43	75.32%
5. Public sector	0.75	0.44	74.68%
**Environmental Information Online Disclosure (NSRD)**	**0.51**	**0.33**	**51.08%**
1. Energy	0.66	0.48	65.58%
2. Buildings and grounds	0.45	0.55	45.45%
3. Purchasing management	0.34	0.48	34.42%
4. Waste management and recycling	0.75	0.44	75.68%
5. Transportation	0.54	0.50	53.90%
6. Food	0.32	0.47	32.47%
**Social Information Online Disclosure (SSRD)**	**0.7**	**0.19**	**70.37%**
1. Summer programs	0.71	0.46	70.78%
2. Employment	0.85	0.36	85.06%
3. Campus services/Student life	0.92	0.27	91.56%
4. Campus safety	0.71	0.45	71.43%
5. Health services	0.81	0.39	81.17%
6. Scholarship	0.81	0.4	80.52%
7. Equal opportunity	0.47	0.5	47.40%
8. Diversity and equity	0.89	0.31	88.96%
9. Disability resources	0.86	0.35	85.71%
10. Statement of integrity	0.14	0.35	14.29%
11. Code of conduct	0.57	0.5	57.14%
**Educational Information Online Disclosure (DSRD)**	**0.65**	**0.31**	**64.72%**
1. Academic	0.64	0.48	63.64%
2. Research	0.49	0.5	48.70%
3. Service	0.82	0.39	81.82%

Source: Own elaboration.

**Table 5:** Descriptive statistics independent variables.

Variable	Minimum	Maximun	Mean	SD
Size	2.26	4.86	4.19	0.53
Affiliation	0.00	1.00	0.38	0.49
Unipriv	0.00	1.00	0.32	0.47
Age	1.58	2.58	2.09	0.21
Ranking position	1.00	154.00	77.50	44.60

Source: Own elaboration.

**Table 6:** Factors underlying the online disclosure of CSR information.

	GSRD	ESRD	NSRD	SSRD	DSRD	
Variable	Standardised Coefficients	Standardised Coefficients	Standardised Coefficients	Standardised Coefficients	Standardised Coefficients	
Size	0.250	0.181	0.301	0.032	0.127	β
	(3.036) **	(2.090)	(3.791) **	(0.476)	(1.457)	t
	95%	95%	99%	−	−	Sig
Affiliation	0.187	−0.003	0.136	−0.112	0.283	β
	(2.190) **	(−0.031)	(1.648)	(−1.633)	(3.367) **	t
	95%	−	−	−	95%	Sig
UniPriv	0.201	−0.082	0.067	−0.129	0.025	β
	(2.351) **	(−0.916)	(0.813)	(−1.877) *	(0.282)	t
	95%	−	−	90%	−	Sig
Age	0.022	0.176	0.004	0.025	0.026	β
	(.269)	(2.039) **	(0.044)	(0.302)	(0.310)	t
	−	95%	−	−	−	Sig
Ranking position	0.206	−0.013	0.326	0.657	0.142	β
	(2.503) **	(−0.150)	(4.097) ***	(9.914) ***	(1.683)	t
	95%	−	99%	99%	−	Sig
F	5.910 ***	2.585 *	8.582 ***	25.161 ***	4.523 **	
R^2^	0.166	0.101	0.225	0.462	0.133	

Source: Own elaboration. Note: * Significant at 0.1; ** Significant at 0.05; *** Significant at 0.01.

## Appendix A

**Table A1:** Universities.

Universities	GSRD	ESRD	NSRD	SSRD	DSRD
1 Harvard	0.40	1.00	0.83	0.45	0.67
2 Calif Berkeley	0.63	0.40	1.00	0.82	0.67
3 Stanford	0.40	0.60	1.00	0.36	1.00
4 Massachusetts Ins Tech	0.47	0.00	0.67	0.18	1.00
5 Calif Inst Tech	0.47	0.80	0.67	0.27	1.00
6 Princenton	0.50	0.80	0.50	0.36	1.00
7 Columbia	0.10	0.80	0.00	0.45	0.67
8 Chicago	0.40	0.80	0.50	0.82	0.67
9 Yale	0.60	1.00	0.83	0.45	0.33
10 Cornell	0.60	1.00	0.83	0.82	1.00
11 Calif Los Angeles	0.50	1.00	1.00	0.64	1.00
12 Calif San Diego	0.50	1.00	0.67	0.64	0.67
13 Pennsylvania	0.40	0.80	1.00	0.36	0.33
14 Washington	0.17	0.00	0.67	0.36	0.33
15 Wisconsin Madison	0.10	0.00	0.50	0.55	1.00
16 Johns Hopkins	0.40	0.00	0.67	0.73	0.67
17 California, S. Fran	0.30	1.00	0.83	0.73	0.00
18 Michigan	0.70	1.00	0.83	0.73	0.33
19 Illinois	0.30	1.00	0.50	0.45	1.00
20 Minnesota	0.50	1.00	0.83	0.55	0.67
21 Northwestern	0.30	1.00	0.83	0.45	0.67
22 Washingt S.Louis	0.37	0.80	0.33	0.82	0.00
23 NY	0.40	0.80	1.00	0.45	0.67
24 Calif Sta Barb	0.40	0.00	0.83	0.55	1.00
25 Colorado B	0.40	0.00	0.67	0.64	0.67
26 Rockefeller	0.40	0.40	0.67	0.18	0.33
27 Duke	0.60	1.00	0.83	0.36	0.67
28 Maryland	0.57	0.00	0.83	0.55	0.67
29 Texas Austin	0.40	0.00	0.17	0.55	0.33
30 North California Chapel	0.20	1.00	0.83	0.55	0.67
31 Pennsyl State	0.20	0.80	0.50	0.82	0.67
32 Calif Davis	0.47	1.00	0.83	1.00	0.67
33 Calif Irvine	0.57	0.00	1.00	0.82	1.00
34 Southern Calif	0.40	0.80	0.83	0.91	1.00
35 Texas Southwestern Medical Center at Dallas	0.00	0.80	0.17	0.73	0.33
36 Vanderbilt	0.40	1.00	1.00	0.64	0.67
37 Rutgers	0.10	1.00	0.67	0.91	0.33
38 Pittsburgh	0.10	1.00	0.50	0.82	0.67
39 Carnegie Mellon	0.27	1.00	0.17	0.55	1.00
40 Ohio State	0.50	1.00	0.67	0.45	0.67
41 Brown	0.40	1.00	0.33	0.64	1.00
42 Florida	0.50	1.00	0.50	0.91	0.67
43 Purdue	0.20	1.00	0.67	0.91	1.00
44 Boston	0.40	0.80	0.83	0.82	0.67
45 Arizona	0.40	1.00	0.50	0.73	0.33
46 Arizona State	0.50	1.00	1.00	0.55	1.00
47 Rochester	0.30	1.00	0.17	0.55	0.00
48 Utah	0.30	1.00	0.50	0.82	0.33
49 Michigan State	0.20	1.00	0.33	0.64	0.67
50 Indiana	0.40	1.00	0.67	0.73	0.67
51 Texas A&M	0.50	1.00	1.00	0.91	1.00
52 Virginia	0.50	1.00	0.67	0.55	1.00
53 Case Western Reserve	0.30	0.80	0.67	0.64	1.00
54 Rice	0.50	0.80	0.67	0.82	1.00
55 Baylor College of Medicine	0.20	0.00	0.33	0.55	0.33
56 Emory	0.40	0.80	0.67	0.73	1.00
57 Georgia Inst	0.40	1.00	0.50	0.64	1.00
58 Mayo Medical School	0.00	0.00	0.00	0.45	0.33
59 North Carolina	0.50	1.00	0.83	0.18	0.67
60 Oregon State	0.37	1.00	1.00	0.82	1.00
61 Georgia	0.40	0.60	0.83	0.91	1.00
62 Tufts	0.40	1.00	1.00	0.91	1.00
63 California, Riverside	0.40	0.00	0.83	0.82	1.00
64 Calif Sta Cruz	0.50	0.00	1.00	0.91	0.67
65 Hawaii at Manoa	0.00	0.00	0.00	0.82	1.00
66 Iowa	0.50	1.00	0.83	0.91	1.00
67 Massachu Amherst	0.10	0.00	0.00	0.64	0.67
68 Massachusetts Medical School-Worcester	0.00	0.00	0.00	0.45	0.33
69 Miami	0.00	1.00	0.00	0.82	0.33
70 Colorado State	0.20	1.00	0.50	1.00	1.00
71 Dartmouth	0.37	1.00	0.33	0.91	0.67
72 Florida State	0.10	1.00	0.17	0.91	0.67
73 George Mason	0.20	1.00	0.67	0.91	0.67
74 Iowa State	0.10	1.00	0.33	0.82	0.67
75 Lousiana State	0.50	1.00	0.67	0.82	0.67
76 Mount Sinai School of Medicine	0.00	0.00	0.00	0.64	0.33
77 Oregon Health and Science University	0.00	0.00	0.00	0.82	0.33
78 State University of New York at Stony Brook	0.20	0.80	0.50	0.55	0.67
79 Alabama at Birmingham	0.50	1.00	0.67	0.91	1.00
80 Connecticut	0.30	1.00	0.67	0.82	0.33
81 Texas Health Science Center at Houston	0.00	1.00	0.00	0.73	0.33
82 Texas M. D. Anderson Cancer Center	0.00	0.80	0.00	0.36	0.33
83 Delaware	0.50	1.00	0.33	0.91	1.00
84 Illinois Chicago	0.37	1.00	0.83	0.91	1.00
85 Maryland, Baltimore	0.20	0.00	0.00	0.55	0.33
86 Nebraska Lincoln	0.10	1.00	0.17	1.00	0.33
87 Tenne knoxv	0.20	1.00	0.17	0.73	0.67
88 Virginia Commonwealth	0.57	1.00	0.83	0.82	1.00
89 Virg Polyt	0.10	1.00	0.33	0.91	0.67
90 Brandeis	0.57	0.80	0.67	0.91	0.67
91 City un NY	0.20	1.00	0.17	0.55	0.33
92 Rensselaer	0.46	0.00	0.67	0.45	0.67
93 Buffalo	0.00	0.00	0.00	0.64	0.00
94 George Washington	0.27	1.00	0.17	0.64	0.67
95 New Mexico	0.20	1.00	0.17	0.73	1.00
96 Texas Health Science Center at San Antonio	0.00	0.00	0.00	0.45	0.33
97 Central Florida	0.47	1.00	0.50	0.82	1.00
98 Cincinnati	0.30	1.00	1.00	0.91	1.00
99 Colorado at Denver	0.40	1.00	0.33	0.82	0.67
100 Houston	0.10	1.00	0.17	0.91	0.33
101 Kansas	0.20	1.00	0.50	0.82	1.00
102 Kentucky	0.30	1.00	0.33	0.73	0.33
103 Medicine and Dentistry New Jersey	0.00	1.00	0.00	0.55	0.00
104 Missouri Columbia	0.47	1.00	0.50	0.73	0.67
105 Notre Dame	0.40	0.80	1.00	0.73	1.00
106 Oregon	0.20	1.00	0.00	0.91	1.00
107 South Carolin	0.10	0.80	0.17	1.00	0.67
108 South Florida	0.50	1.00	0.67	0.55	1.00
109 Vermont	0.20	1.00	0.50	0.91	0.33
110 Wash State Pullman	0.40	0.00	0.83	0.82	0.67
111 Yeshiva	0.40	0.00	0.67	0.36	0.33
112 Brigham Young	0.30	0.00	0.67	0.55	0.67
113 Clemson	0.10	1.00	0.50	0.64	0.33
114 Drexel	0.20	0.80	0.17	0.73	0.00
115 Georgetown	0.50	1.00	0.50	0.82	1.00
116 Kansas State	0.40	1.00	0.67	0.82	1.00
117 Medical South Carolina	0.37	1.00	0.50	0.91	0.33
118 Saint Louis	0.20	0.00	0.00	0.82	0.67
119 San Diego State	0.20	0.00	0.67	0.64	0.33
120 State N. Y. Albany	0.40	0.00	0.67	0.82	0.67
121 State New York Health Science Center at Brooklyn	0.10	0.00	0.17	0.45	0.33
122 Syracuse	0.40	0.80	0.50	0.91	0.67
123 Temple	0.17	0.80	0.83	0.73	1.00
124 Texas Tech	0.00	0.00	0.00	0.64	0.33
125 Montana - Missoula	0.20	0.80	0.67	0.91	1.00
126 Texas at Dallas	0.20	1.00	0.00	0.73	0.33
127 Texas Medical Branch at Galveston	0.10	0.00	0.33	0.55	0.33
128 Thomas Jefferson	0.00	0.00	0.00	0.45	0.33
129 Tulane	0.10	0.80	0.00	0.91	0.67
130 Alaska-Fairbanks	0.50	0.00	1.00	0.91	1.00
131 Arkansas at Fayetteville	0.57	1.00	0.33	0.82	1.00
132 Nevada-Reno	0.57	1.00	0.33	0.91	0.33
133 New Hampshire-Durham	0.10	1.00	0.00	0.91	0.67
134 Oklahoma	0.30	1.00	0.50	0.91	0.33
135 Rhode Island	0.57	1.00	0.50	0.91	1.00
136 Wayne State	0.50	1.00	1.00	0.82	0.67
137 Wake Forest	0.40	0.00	0.50	0.91	0.33
138 Auburn	0.40	0.00	0.67	0.36	0.00
139 Boston College	0.50	0.80	0.50	0.91	1.00
140 Indiana University-Purdue Indianapolis	0.57	0.00	0.50	0.82	0.67
141 Kent State	0.20	1.00	0.17	0.82	0.33
142 Lehigh	0.30	0.00	0.00	0.91	0.33
143 Medical College of Wisconsin	0.20	1.00	0.00	0.36	0.00
144 Montana State Bozeman	0.40	1.00	0.67	0.91	1.00
145 Northeastern	0.40	0.80	0.83	0.82	0.33
146 Ohio	0.30	1.00	0.83	0.82	1.00
147 Portland State	0.30	0.00	1.00	0.82	1.00
148 Connecticut Health Center	0.20	1.00	0.50	0.55	0.33
149 Texas at San Antonio	0.20	1.00	0.33	0.45	0.00
150 Arkansas at Little Rock	0.40	1.00	0.83	0.82	1.00
151 Kansas Medical Center	0.00	1.00	0.00	0.73	0.33
152 Nebraska Medical Center	0.00	1.00	0.00	0.64	0.33
153 Wyoming	0.57	1.00	0.33	0.64	1.00
154 Utah State	0.40	1.00	0.33	0.82	1.00

## References

[1] European Commission A Renewed EU Strategy 2011–14 for Corporate Social Responsibility 2011. http://eur-lex.europa.eu/LexUriServ/LexUriServ.do?uri_COM:2011:0681:FIN:EN:PDF.

[2] Zhang Q., Oo B.L., Lim B.T.H. (2019). Drivers, motivations, and barriers to the implementation of corporate social responsibility practices by construction enterprises: A review. J. Clean. Prod..

[3] Rodríguez Bolívar M.P., Garde Sánchez R., López Hernández A.M. (2013). Online disclosure of corporate social responsibility information in leading Anglo-American universities. J. Environ. Pol. Plan..

[4] Shelomentsev A.G., Kozlova A.O., Doroshenko V., Stanislavovna K.E. (2017). The leading universities’ social responsibility and regional development. Eur. Proc. Soc. Behav. Sci..

[5] Garde-Sánchez R., Bolívar M.P.R., López-Hernández A.M. (2013). Online disclosure of university social responsibility: A comparative study of public and private US universities. Environ. Educ. Res..

[6] Jorge M.L., Peña F.J.A. (2014). Determinants of corporate social responsibility and business ethics education in Spanish universities. Bus. Ethic. Eur. Rev..

[7] Belal A.R., Cooper S. (2011). The absence of corporate social responsibility reporting in Bangladesh. Crit. Perspect. Account..

[8] Campopiano G., De Massis A. (2015). Corporate social responsibility reporting: A content analysis in family and non-family firms. J. Bus. Ethics.

[9] Hahn R., Kühnen M. (2013). Determinants of sustainability reporting: A review of results, trends, theory, and opportunities in an expanding field of research. J. Clean. Prod..

[10] Daub C.-H. (2007). Assessing the quality of sustainability reporting: An alternative methodological approach. J. Clean. Prod..

[11] Sassen R., Azizi L. (2017). Voluntary disclosure of sustainability reports by Canadian universities. J. Bus. Econ..

[12] Roberts R.W. (1992). Determinants of corporate social responsibility disclosure: An application of stakeholder theory. Account. Organ. Soc..

[13] Chen J.C., Roberts R.W. (2010). Toward a more coherent understanding of the organization-society relationship: A theoretical consideration for social and environmental accounting research. J. Bus. Ethics.

[14] Vaz N., Fernandez-Feijoo B., Ruiz S. (2016). Integrated reporting: An international overview. Bus. Ethic. Eur. Rev..

[15] Freeman R.E. (1984). Strategic Management: A Stakeholder Approach.

[16] Smith J., Haniffa R., Fairbrass J. (2010). A conceptual framework for investigating ‘capture’ in corporate sustainability reporting assurance. J. Bus. Ethics.

[17] Frynas J.G., Yamahaki C. (2016). Corporate social responsibility: Review and roadmap of theoretical perspectives. Bus. Ethic. Eur. Rev..

[18] Chiu T.-K., Wang Y.-H. (2015). Determinants of social disclosure quality in Taiwan: An application of stakeholder theory. J. Bus. Ethics.

[19] Boesso G., Kumar K. (2007). Drivers of corporate voluntary disclosure: A framework and empirical evidence from Italy and the United States. Account. Audit. Accoun..

[20] Hooghiemstra R. (2000). Corporate communication and impression management: New perspectives on why companies engage in corporate social reporting. J. Bus. Ethics.

[21] Cormier D., Gordon I.M. (2001). An examination of social and environmental reporting strategies. Account. Audit. Account. J..

[22] Caba C., López A., Rodríguez M.P. (2005). Citizens’ access to on-line governmental financial information: Practices in the European Union countries. Gov. Inform. Q..

[23] Castelo B.M., Lima R.L. (2008). Factors influencing social responsibility disclosure by Portuguese companies. J. Bus. Ethics.

[24] Reverte C. (2008). Determinants of corporate social responsibility disclosure ratings by Spanish listed firms. J. Bus. Ethics.

[25] Gordon T., Fischer M., Malone D., Tower G. (2002). A comparative empirical examination of extent of disclosure by private and public colleges and universities in the United States. J. Account. Public Policy.

[26] Gallego-Álvarez I., Rodríguez-Domínguez L., García-Sánchez I. (2011). Information disclosed online by Spanish universities: Content and explanatory factors. Online Inf. Rev..

[27] Monteiro S., Aibar-Guzmán B. (2009). Determinants of environmental disclosure in the annual reports of large companies operating in Portugal. Corp. Soc. Responsib. Environ. Manag..

[28] Opoku R., Caruana A., Pitt L., Berthon P. (2009). Online communication of brand personality: A study of MBA programs of top business schools. J. Gen. Manag..

[29] Schimmel K., Motley D., Racic S., Marco G., Eschenfelder M. (2010). The importance of university web pages in selecting a higher education institution. Res. High. Educ..

[30] Kastenholz E., Ladero M.D.L.M.G., Galera-Casquet C., Valero-Amaro V. (2004). La responsabilidad social en las entidades financieras: Un estudio exploratorio de la situación en Portugal. Int. Rev. Public Nonprofit Mark..

[31] Gray R., Kouhy R., Lavers S. (1995). Corporate social and environmental reporting: A review of the literature and a longitudinal study of UK disclosure. Account. Audit. Accoun..

[32] Jones K. (2001). Study on Environmental Reporting by Companies.

[33] Donaldson T., Preston L.E. (1995). The stakeholder theory of the corporation: Concepts, evidence, and implications. Acad. Manag. Rev..

[34] Murias P., De Miguel J.C., Rodríguez D. (2008). A composite indicator for university quality assessment: The case of the Spanish higher education system. Soc. Indic. Res..

[35] Cooke T.E. (1989). Voluntary Corporate Disclosure by Swedish Companies. J. Int. Financial Manag. Account..

[36] Robb S.W.G., Single L.E., Zarzeski M.T. (2001). Nonfinancial disclosures across Anglo-American countries. J. Int. Account. Audit. Tax..

[37] Link A.N., Siegel D.S., Bozeman B. (2007). An empirical analysis of the propensity of academics to engage in informal university technology transfer. Ind. Corp. Chang..

[38] Hicks D. (2012). Performance-based university research funding systems. Res. Policy.

[39] Cattaneo M., Meoli M., Signori A. (2014). Performance-based funding and university research productivity: The moderating effect of university legitimacy. J. Technol. Transf..

[40] McNamara K.H. (2010). Research article: Fostering sustainability in higher education: A mixed-methods study of transformative leadership and change strategies. Environ. Pract..

[41] Lozano R., Ceulemans K., Alonso-Almeida M., Huisingh D., Lozano F.J., Waas T., Lambrechts W., Lukman R., Hugé J. (2015). A review of commitment and implementation of sustainable development in higher education: Results from a worldwide survey. J. Clean. Prod..

[42] Lee K.-H., Barker M., Mouasher A. (2013). Is it even espoused? An exploratory study of commitment to sustainability as evidenced in vision, mission, and graduate attribute statements in Australian universities. J. Clean. Prod..

[43] Huang S.K., Wang Y.-L. (2013). A comparative study of sustainability management education in China and the USA. Environ. Educ. Res..

[44] Cormier D., Gordon I.M., Magnan M. (2004). Corporate environmental disclosure: Contrasting management’s perceptions with reality. J. Bus. Ethics.

[45] UNESCO (2005). UN decade of education for sustainable development 2005–2014. International Implementation Scheme.

[46] Decter M.H. (2009). Comparative review of UK-USA industry-university relationships. Educ. Train..

[47] Billaut J.C.J., Bouyssou D., Vincke P. (2010). Should you believe in the Shanghai ranking?. Science.

[48] Buela-Casal G., Gutiérrez-Martínez O., Bermúdez-Sánchez M.P., Vadillo-Muñoz O. (2007). Comparative study of international academic rankings of universities. Science.

[49] Leydesdorff L., Shin J.C. (2011). How to evaluate universities in terms of their relative citation impacts: Fractional counting of citations and the normalization of differences among disciplines. J. Am. Soc. Inf. Sci. Technol..

[50] Docampo D. (2008). International rankings and quality of the university systems. Rev. Educ. Madr. Spec. Issue.

[51] Kuzey C., Uyar A. (2017). Determinants of sustainability reporting and its impact on firm value: Evidence from the emerging market of Turkey. J. Clean. Prod..

[52] Jöreskog K.G., Sörbom D. (1996). LISREL VIII..

[53] Fonseca A., Macdonald A., Dandy E., Valenti P. (2011). The state of sustainability reporting at Canadian universities. Int. J. Sustain. High. Educ..

[54] Yuan X., Zuo J., Huisingh D. (2013). Green universities in China-what matters?. J. Clean. Prod..

[55] Global Reporting Initiative (GRI) (2002). Sustainability Reporting Guidelines.

[56] Global Reporting Initiative (GRI) (2013). G4 Sustainability Reporting Guidelines: Reporting Principles and Standard Disclosure.

[57] Lozano R. (2006). A tool for a Graphical Assessment of Sustainability in Universities (GASU). J. Clean. Prod..

[58] Cole L. (2003). Assessing Sustainability on Canadian University Campuses: Development of a Campus Sustainability Assessment Framework. Master’s Thesis.

[59] Lozano R. (2011). The state of sustainability reporting in universities. Int. J. Sustain. High. Educ..

[60] AASHE (2010). Sustainability Tracking, Assessment & Rating System: Version 1.0 Technical Manual.

[61] Jones K., Alabaster T., Walton J. (1998). Virtual environments for environmental reporting. Greener Manag. Int..

[62] Ho P.-L., Tower G., Barako D.G. (2008). Improving governance leads to improved corporate communication. Corp. Ownersh. Control..

[63] Nicolaides A. (2006). The implementation of environmental management towards sustainable universities and education for sustainable development as an ethical imperative. Int. J. Sustain. High. Educ..

[64] Wohlrabe K., De Moya-Anegon F., Bornmann L. (2019). How efficiently do elite US universities produce highly cited papers?. Publications.

